# Association between perceived usefulness of generative AI for learning and generative AI dependency among university students in Changde, China: a cross-sectional study of statistical indirect effects

**DOI:** 10.3389/fpsyg.2026.1859317

**Published:** 2026-05-29

**Authors:** Yan Liu, Yuanbing Liu

**Affiliations:** 1 School of Information, Changde College, Changde, Hunan, China; 2 School of Continuing Education, Hunan University of Arts and Science, Changde, Hunan, China

**Keywords:** academic procrastination, generative AI dependency, metacognitive self-regulation, perceived usefulness of generative AI for learning, statistical indirect effects, university students

## Abstract

**Background:**

As generative AI becomes increasingly embedded in university learning, whether greater perceived usefulness of generative AI for learning is associated with greater generative AI dependency remains unclear, as do the potential statistical indirect roles of metacognitive self-regulation and academic procrastination.

**Methods:**

This cross-sectional questionnaire study surveyed 1,869 university students from three higher education institutions in Changde, Hunan Province, China. Eligible participants were aged 18 years or older and had used generative AI to assist learning within the past month. Pearson correlations were used to examine bivariate associations, and regression and PROCESS Model 6 analyses were conducted after controlling for sex, age, and grade to estimate specific and serial statistical indirect effects. Statistical indirect effects were tested using 5,000 bootstrap resamples and 95% confidence intervals (CIs).

**Results:**

Perceived usefulness of generative AI for learning was positively associated with generative AI dependency (total association: *β* = 0.303, *p* < 0.001). It was negatively associated with metacognitive self-regulation (*β* = −0.260, *p* < 0.001) and positively associated with academic procrastination (*β* = 0.254, *p* < 0.001). Metacognitive self-regulation was negatively associated with academic procrastination (*β* = −0.218, *p* < 0.001) and generative AI dependency (*β* = −0.154, *p* < 0.001), whereas academic procrastination was positively associated with generative AI dependency (*β* = 0.224, *p* < 0.001). After metacognitive self-regulation and academic procrastination were entered, the direct association remained significant (*β* = 0.194, *p* < 0.001). The unstandardized statistical indirect effects via metacognitive self-regulation, academic procrastination, and the serial path involving both variables were 0.035 (95% CI [0.0236, 0.0479]), 0.050 (95% CI [0.0374, 0.0635]), and 0.011 (95% CI [0.0078, 0.0150]), respectively; the total statistical indirect effect was 0.096 (95% CI [0.0787, 0.1150]).

**Conclusion:**

Higher perceived usefulness of generative AI for learning was statistically associated with higher generative AI dependency, with statistical indirect effects involving lower metacognitive self-regulation and higher academic procrastination. Given the cross-sectional design, PROCESS Model 6 was used to estimate specific and serial statistical indirect effects, which should not be interpreted as evidence that temporal ordering has been established or that causal mechanisms have been confirmed.

## 1 Introduction

As generative AI has rapidly entered higher education, its convenience in information retrieval, text organization, assignment support, and task progression has drawn increasing attention. In 2025, the Ministry of Education and eight other departments jointly issued the Opinions on Accelerating the Advancement of Education Digitalization, emphasizing AI-enabled educational transformation and providing an important policy context for the growing use of generative AI in university learning (Ministry of Education of the People’s Republic of China et al., 2025). Beyond these benefits, however, continued use of generative AI in learning has also raised concerns about potential risks. Commentary has suggested that generative AI dependency is emerging as an issue warranting attention (Kooli et al., 2025), and preliminary empirical evidence indicates that university students may show persistent reliance on generative AI, dependency tendencies, and possible academic costs associated with such use (Revesai, 2025). Accordingly, examining the association between perceived usefulness of generative AI for learning and dependency-oriented use is important for understanding students’ learning regulation and dependency-related behavior in AI-supported higher education.

From the perspective of technology acceptance research, perceived usefulness or performance expectancy is a key cognitive basis associated with continued technology use (Venkatesh et al., 2003). In the literature on problematic internet use, positive outcome expectancy has likewise been viewed as a proximal cognitive factor associated with dependency risk (Lin et al., 2018). Prior research has also suggested that utility beliefs and habitual use may be jointly associated with addictive tendencies (Paiman and Fauzi, 2024). In this study, generative AI dependency is distinguished from technology overuse and continuance intention because it emphasizes persistent reliance on generative AI in learning, difficulty reducing such reliance, and possible cognitive or academic costs. In learning contexts, stronger perceived usefulness of generative AI may coincide not only with greater routine incorporation of the tool, but also with reduced learning monitoring, delayed task progression, and a stronger tendency toward dependency-oriented use. However, existing research still lacks a systematic examination of perceived usefulness of generative AI for learning, metacognitive self-regulation, academic procrastination, and generative AI dependency within a single educational psychology framework. This study therefore provides an integrative and context-specific contribution by linking technology-related learning beliefs with self-regulated learning, academic procrastination, and dependency-oriented generative AI use. Accordingly, this study aimed to (1) examine the association between perceived usefulness of generative AI for learning and generative AI dependency; (2) investigate the independent and serial statistical indirect effects involving metacognitive self-regulation and academic procrastination in this association; and (3) examine a theory-informed framework of statistical associations among these variables. Given the cross-sectional questionnaire design, the proposed paths were specified to characterize statistical associations consistent with the hypothesized theoretical ordering and were not intended to establish temporal order or causal mechanisms.

## 2 Literature review and research hypotheses

### 2.1 Perceived usefulness of generative AI for learning and generative AI dependency

In technology acceptance research, perceived usefulness or performance expectancy refers to individuals’ subjective judgment of whether a technology can improve task efficiency, task quality, and performance, and it is widely regarded as an important cognitive basis associated with continued use (Venkatesh et al., 2003). In the present study, perceived usefulness of generative AI for learning refers to students’ overall evaluation of the value of generative AI for information retrieval, content organization, task completion, and learning support. By contrast, generative AI dependency differs from both technology overuse and continuance intention. Technology overuse mainly emphasizes excessive frequency, duration, or intensity of use, whereas continuance intention concerns willingness to keep using a technology. Generative AI dependency in learning contexts instead emphasizes persistent reliance on generative AI for cognitive processing, task completion, and learning decisions, difficulty reducing such reliance, and possible cognitive and academic costs. Existing studies and commentary suggest that generative AI dependency is an emerging issue worthy of attention among university students (Kooli et al., 2025; Revesai, 2025). Theoretically, students who more strongly perceive generative AI as capable of saving time, improving learning efficiency, and enhancing task outcomes may be more likely to incorporate it into routine learning and show dependency-oriented reliance. Consistent with this view, research on problematic internet use indicates that positive outcome expectancy is an important proximal cognitive factor associated with dependency risk (Lin et al., 2018). Studies integrating technology acceptance and usage habit further suggest that utility beliefs and habitual use may be associated with addictive or dependency-oriented tendencies (Paiman and Fauzi, 2024; Pitafi et al., 2020). Therefore, higher perceived usefulness of generative AI for learning may be associated with a stronger tendency toward generative AI dependency. Hypothesis 1: Perceived usefulness of generative AI for learning is positively associated with generative AI dependency.

### 2.2 Statistical indirect role of metacognitive self-regulation

Metacognitive self-regulation may be involved in a statistical indirect association between perceived usefulness of generative AI for learning and generative AI dependency. It refers to learners’ capacity to set goals, monitor ongoing processes, regulate strategies, and reflect on outcomes during learning, and it is an important component of self-regulated learning. Prior research has found that students with executive function difficulties are more likely to perceive generative AI as useful for academic tasks, suggesting that perceived usefulness and self-regulatory resources are not entirely independent (Klarin et al., 2024). More broadly, research on digital tool use suggests that highly convenient and readily accessible technologies are often accompanied by cognitive offloading, whereby learners shift part of the processing burden to external systems and may invest less in analysis, memory, and monitoring (Barr et al., 2015; Firth et al., 2019; Sparrow et al., 2011). Research on trust in automation and adjacent fields indicates that when automated systems are trusted and verification is costly, users may engage in less checking and more direct acceptance of outputs; this pattern has been discussed in studies of automation bias, complacency, AI trust, and security automation (Gerlich, 2024; Lyell and Coiera, 2017; Merritt et al., 2019; Parasuraman et al., 1993; Skitka et al., 1999; Tilbury and Flowerday, 2024). Accordingly, higher perceived usefulness of generative AI for learning may be associated with lower metacognitive self-regulation.

Metacognitive self-regulation may also be associated with generative AI dependency. Prior research has shown that insufficient self-regulation is associated with problematic digital use, including smartphone addiction, suggesting that weaker planning, behavioral control, and use boundaries may coexist with dependency-oriented tendencies (Mahapatra, 2019). Evidence from problematic social networking use further suggests that problematic use is linked to weaker reflective/control processes and impulsivity, although the specific roles of executive functions and inhibitory control may vary across studies (Turel and Qahri-Saremi, 2016; Wegmann et al., 2020). In learning contexts, lower metacognitive self-regulation may be associated with default reliance on generative AI, frequent help seeking, and dependency-oriented use. Hypothesis 2: Metacognitive self-regulation is involved in a statistical indirect path linking perceived usefulness of generative AI for learning and generative AI dependency.

### 2.3 Statistical indirect role of academic procrastination

Academic procrastination generally refers to unnecessary delay in assignments, exam preparation, course projects, and other academic tasks, and it is often regarded as a form of self-regulation failure (Steel and Klingsieck, 2016). Existing research suggests that procrastination is not merely a byproduct of poor academic outcomes; it may also be involved in the association between digital distraction and negative academic outcomes (Jin et al., 2024). In generative AI-supported learning contexts, students’ perceptions of the tool as time-saving, effort-saving, and capable of producing rapid outputs may be statistically associated with academic procrastination, which may represent a proximal behavioral correlate in the association between perceived usefulness and dependency-oriented use. From the perspective of behavioral economics, present bias suggests that individuals overweight immediate rewards and discount delayed costs, and related field evidence shows that students with stronger present-bias tendencies are more likely to delay task completion (Bisin and Hyndman, 2020). Research on digital media use likewise suggests that low-cost, immediately rewarding, and habitually accessible tools may shift from supportive resources to “procrastination tools” (Meier et al., 2016). Accordingly, when generative AI is perceived as an efficient and readily available learning resource, such perceptions may be associated with delayed task initiation, delayed task progression, and higher academic procrastination.

Academic procrastination also shows a relatively consistent association with dependency-oriented digital use. Research on university students has found that academic procrastination is positively associated with internet addiction and explains variance in it (Nadarajan et al., 2023). Although some studies suggest that problematic digital use may also be associated with procrastination through weaker core self-evaluation and self-control (Geng et al., 2018), within the theoretical framework of the present study, academic procrastination is conceptualized as a proximal behavioral component of the statistical indirect path linking perceived usefulness of generative AI for learning and generative AI dependency. Taken together, higher perceived usefulness of generative AI for learning may be associated with higher academic procrastination, and higher academic procrastination may be associated with higher generative AI dependency. Hypothesis 3: Academic procrastination is involved in a statistical indirect path linking perceived usefulness of generative AI for learning and generative AI dependency.

### 2.4 Serial statistical indirect effect of metacognitive self-regulation and academic procrastination

In addition to the two independent statistical indirect pathways, existing research suggests that metacognitive self-regulation and academic procrastination may jointly form a serial statistical indirect pathway linking perceived usefulness of generative AI for learning and generative AI dependency. Prior research has noted that learners may externalize the use of digital tools not only because such tools are available, but also because they reduce immediate cognitive load and provide rapid, low-cost task support. Under conditions of high convenience, high cognitive load, or high information value, such tool use may coincide with reduced deep processing and diminished investment in self-monitoring (Skulmowski, 2023). Accordingly, in generative AI-supported learning contexts, when students more strongly perceive the tool as efficient, convenient, and readily available, they may be more likely to delegate part of the planning, checking, and judgment work that would otherwise be performed internally to an external system, which may coincide with lower levels of metacognitive self-regulation. Metacognitive self-regulation was specified before academic procrastination because it reflects relatively upstream learning processes, such as planning, monitoring, and strategy regulation, whereas academic procrastination reflects downstream delay in task initiation and task execution. Research on self-regulated learning also suggests that metacognitive regulation is closely tied to study planning, task progression, and behavioral adjustment, whereas lower academic self-regulation is more likely to co-occur with procrastinatory behavior (Ragusa et al., 2023; Balkis and Duru, 2016). Therefore, perceived usefulness of generative AI for learning may be associated with lower metacognitive self-regulation, which may in turn be associated with higher academic procrastination.

**Figure 1:**
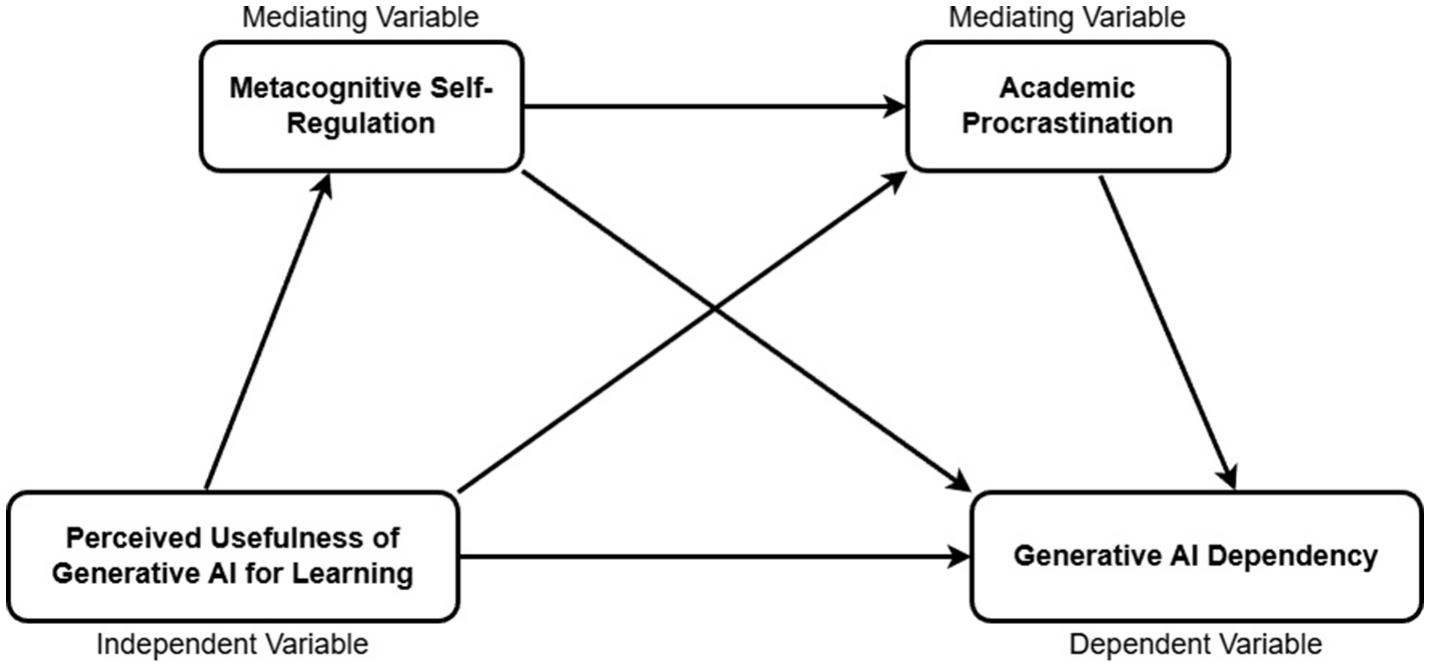
Theory-informed statistical indirect association model. The figure depicts the hypothesized statistical associations among perceived usefulness of generative AI for learning, metacognitive self-regulation, academic procrastination, and generative AI dependency. Arrows represent theory-informed statistical associations rather than established temporal or causal effects.

Academic procrastination may then be associated with higher levels of dependency-oriented digital use. Existing research suggests that digital tools may be incorporated into procrastinatory use as convenient resources for avoiding task demands, relieving immediate stress, or delaying confrontation with academic challenges (Meier et al., 2016). In generative AI-supported learning contexts, when students experience delayed task initiation, accumulating time pressure, and increasing need for last-minute remediation, generative AI may be more readily viewed as a form of compensatory support and thus be associated with higher generative AI dependency. Network-analytic evidence from smartphone addiction and academic procrastination likewise suggests coupling at more fine-grained behavioral or symptom levels (Song et al., 2025). At the same time, cross-sectional evidence also suggests that problematic digital use and procrastination may be related in reverse or cyclical ways (Qi et al., 2025). Therefore, the serial ordering examined in the present study is theory-driven and is intended to test a statistical pathway consistent with the existing literature rather than to establish temporal precedence or causal direction. Taken together, perceived usefulness of generative AI for learning may be associated with lower metacognitive self-regulation, lower metacognitive self-regulation may in turn be associated with higher academic procrastination, and higher academic procrastination may be associated with higher generative AI dependency. Hypothesis 4: Metacognitive self-regulation and academic procrastination jointly form a serial statistical indirect path linking perceived usefulness of generative AI for learning and generative AI dependency (Figure 1).

In summary, previous research suggests that perceived usefulness of generative AI for learning may be associated with generative AI dependency and that metacognitive self-regulation and academic procrastination may play independent and sequential statistical roles in this association. Based on this literature, the present study further examined (1) the association between perceived usefulness of generative AI for learning and generative AI dependency; (2) the statistical indirect role of metacognitive self-regulation in this association; (3) the statistical indirect role of academic procrastination in this association; and (4) the serial statistical indirect effect involving metacognitive self-regulation and academic procrastination.

## 3 Research subjects and methods

### 3.1 Participants

This cross-sectional questionnaire survey was conducted from March 10 to March 20, 2026, among undergraduate and postgraduate students from selected classes at three higher education institutions in Changde, Hunan Province, China. Anonymous online questionnaires were distributed via Wenjuanxing during students’ after-class time, with the assistance of teachers from the selected classes, and participation was entirely voluntary. The inclusion criteria were as follows: (1) being a currently enrolled undergraduate or postgraduate student at one of the three participating institutions; (2) being 18 years of age or older; (3) having used generative AI tools to assist learning within the past month; and (4) providing online informed consent before participation. The exclusion criteria were as follows: (1) failure to provide informed consent; (2) incomplete questionnaire responses or substantial missing data on key variables; and (3) an abnormally short completion time (less than 3 min), which was treated as indicating invalid or insufficiently engaged responding given the expected questionnaire length. A total of 1,900 questionnaires were distributed. After data screening, 1,869 valid questionnaires were retained, yielding a valid questionnaire retention rate of 98.37%. Among the valid participants, 860 were male (46.0%) and 1,009 were female (54.0%). In terms of age group, 273 participants were 18 years old (14.6%), 484 were 19 years old (25.9%), 563 were 20 years old (30.1%), and 549 were aged 21 years or older (29.4%). In terms of grade, 372 were first-year undergraduates (19.9%), 458 were second-year undergraduates (24.5%), 484 were third-year undergraduates (25.9%), 368 were fourth-year undergraduates (19.7%), and 187 were postgraduate students (10.0%).

The study protocol was approved by the Ethics Review Committee of Changde College (Approval No. CDXY 26–0311-01), and the study was conducted in accordance with the Declaration of Helsinki. Before entering the formal questionnaire, participants first read the online informed consent form and could proceed only after indicating their voluntary agreement to participate. The questionnaire was completed anonymously, no personally identifiable information was collected, and the data were used only for academic research and reported in aggregate form.

### 3.2 Measures

#### 3.2.1 Perceived usefulness of generative AI for learning

This study used the Chinese version of the perceived usefulness subscale of the TAME-ChatGPT Usage Scale to assess perceived usefulness of generative AI for learning among university students (Sallam et al., 2023, 2024). To reflect the multi-tool learning environment in Chinese higher education, participants were first asked to identify the generative AI tool they had used most frequently for learning during the past month and then answered the subsequent items with that tool in mind. In the Chinese item wording, “generative AI tool/AI” referred to the selected tool. The subscale contains six items; an original example item is “ChatGPT helps me to save time when searching for information.” In the adapted Chinese wording, “ChatGPT” was replaced by “generative AI tool/AI,” which referred to the selected tool. Items were rated on a 5-point Likert scale ranging from 1 = strongly disagree to 5 = strongly agree. No reverse-coded items were included. The mean of the six items was used as the total score, with higher scores indicating greater perceived usefulness of generative AI for learning. In the present sample (*N* = 1,869), Cronbach’s alpha was 0.876, and model fit was good, *χ*^2^/df = 2.817, RMSEA = 0.031, CFI = 0.997, and TLI = 0.994. Because contextual anchoring and adapted item wording were used for a multi-tool learning environment, the resulting scores should not be directly compared with findings from studies focused on a single tool.

#### 3.2.2 Metacognitive self-regulation

This study used the Chinese version of the Metacognitive Self-Regulation subscale from the Motivated Strategies for Learning Questionnaire to assess metacognitive self-regulation among university students (Pintrich et al., 1991, 1993). The subscale contains 12 items and primarily assesses planning, monitoring, regulation, and reflection during learning. To provide course-based contextual anchoring, participants were instructed to answer with reference to the course to which they had devoted the most time or considered the most important during the current semester. An example item is “I ask myself questions to make sure I understand the material I have been studying in this class.” The original scale used a 7-point Likert format; to maintain a consistent response format across the questionnaire and reduce respondent burden, this study adopted a 5-point Likert scale ranging from 1 = not at all true of me to 5 = very true of me. Two negatively worded items were reverse coded before analysis. The mean of the 12 items was used as the total score, with higher scores indicating higher metacognitive self-regulation. In the present sample (*N* = 1,869), Cronbach’s alpha was 0.923, and model fit was good, *χ*^2^/df = 1.667, RMSEA = 0.019, CFI = 0.997, and TLI = 0.996. Because the response format was modified and course-based contextual anchoring was used, the resulting scores should not be directly compared with findings based on the original 7-point format or different contextual instructions.

#### 3.2.3 Academic procrastination

This study used the Chinese version of the Academic Procrastination Scale-Short Form to assess academic procrastination among university students (Yockey, 2016). This unidimensional short form contains five items assessing delay tendencies in assignments, exam preparation, course projects, and other deadline-based academic tasks. To maintain a consistent time frame across the questionnaire, participants were instructed to respond based on their general learning activities during the past month. An example item is “I put off projects until the last minute.” Items were rated on a 5-point Likert scale ranging from 1 = disagree to 5 = agree. The mean of the five items was used as the total score, with higher scores indicating a stronger tendency toward academic procrastination. In the present sample (*N* = 1,869), Cronbach’s alpha was 0.859, and model fit was good, *χ*^2^/df = 1.506, RMSEA = 0.016, CFI = 0.999, and TLI = 0.999. Because a past-month time frame was used to maintain consistency across the questionnaire, the resulting scores should be interpreted in relation to this specified time frame.

#### 3.2.4 Generative AI dependency

This study used the Chinese version of the Generative AI Dependency Scale to assess generative AI dependency among university students (Goh et al., 2025). The scale includes three dimensions—cognitive distress, negative consequences, and withdrawal reactions—and a total of 11 items assessing individuals’ dependency-oriented or problematic-use tendencies regarding generative AI. Because Chinese university students may use multiple generative AI tools in learning and information processing, participants were first asked to identify the generative AI tool they had used most frequently during the past month and then completed the subsequent items with that tool in mind. An example item is “I get frustrated or irritable when I am unable to use generative AI,” with “generative AI” referring to the selected tool in the adapted response context. Items were rated on a 5-point Likert scale ranging from 1 = strongly disagree to 5 = strongly agree. Mean scores were calculated for the three dimensions, and the mean of all 11 items was used as the overall score in the subsequent analyses, with higher scores indicating greater generative AI dependency. In the present sample (*N* = 1,869), Cronbach’s alpha values for cognitive distress, negative consequences, and withdrawal reactions were 0.793, 0.822, and 0.813, respectively, and model fit was good, *χ*^2^/df = 1.782, RMSEA = 0.020, CFI = 0.996, and TLI = 0.995. Because responses were anchored to the selected tool in a specific tool-use situation, the resulting scores should not be directly compared with findings from studies that did not restrict responses to a specific tool context. In the present study, the scale was used only as a continuous indicator of dependency-oriented tendency and not for diagnostic or categorical interpretation.

### 3.3 Statistical analysis

Data were analyzed using SPSS 26.0 and AMOS. The analyses involved two components: measurement evaluation and examination of associations among variables. For measurement evaluation, Cronbach’s alpha was used to assess internal consistency, confirmatory factor analysis was conducted to examine the fit of the measurement model, and common method bias was assessed using Harman’s single-factor test and an additional CFA-based comparison between the four-factor and one-factor CFA models. After measurement evaluation, composite mean scores were used in the subsequent regression and PROCESS analyses. For the examination of associations among variables, descriptive statistics were used to summarize sample characteristics and the distributions of the main variables, independent-samples t tests and one-way analyses of variance were used to test group differences by sex, age, and grade, respectively, and Pearson correlations were used to examine bivariate associations among the main variables. In the regression and PROCESS analyses, sex, age, and grade were included as covariates. PROCESS Model 6 was used to estimate the direct association, the two specific statistical indirect effects through metacognitive self-regulation and academic procrastination, and the serial statistical indirect effect involving these two variables; given the cross-sectional design, these estimates were not interpreted as evidence of causal mediation or temporal ordering. Indirect effects were estimated using 5,000 bootstrap resamples, and 95% confidence intervals that did not include zero were taken to indicate statistical significance. All tests were two-tailed, and statistical significance was set at *p* < 0.05.

## 4 Results

### 4.1 Perceived usefulness of generative AI for learning, metacognitive self-regulation, academic procrastination, and generative AI dependency

Table 1 shows that, in the overall sample (*N* = 1,869), the mean scores (M ± SD) of Perceived Usefulness of Generative AI for Learning (PUGAIL), Metacognitive Self-Regulation (MSR), Academic Procrastination (AP), and Generative AI Dependency (GAID) were 3.54 ± 0.69, 3.36 ± 0.66, 3.63 ± 0.69, and 3.46 ± 0.61, respectively. Group difference analyses showed significant sex differences across all four variables, with female students scoring higher than male students on PUGAIL, MSR, AP, and GAID (all *p* < 0.05). One-way analyses of variance by age group and grade level also showed significant between-group differences for all four variables (all *p* < 0.05). Accordingly, sex, age, and grade were included as covariates in the subsequent regression and PROCESS analyses.

**Table 1:** Descriptive statistics (M ± SD) and group difference tests for PUGAIL, MSR, AP, and GAID.

Group	*N*	Perceived usefulness of generative AI for learning	Metacognitive self-regulation	Academic procrastination	Generative AI dependency
Male	860	3.49 ± 0.69	3.33 ± 0.66	3.58 ± 0.68	3.42 ± 0.60
Female	1,009	3.59 ± 0.68	3.39 ± 0.66	3.68 ± 0.70	3.50 ± 0.61
Overall	1869	3.54 ± 0.69	3.36 ± 0.66	3.63 ± 0.69	3.46 ± 0.61
Sex differences (*t*)		2.972**	2.015*	3.085**	2.728**
Age differences (*F*)		5.301**	4.469**	4.868**	2.744*
Grade differences (*F*)		4.137**	4.637**	3.723**	3.470**

Values are presented as M ± SD. Sex differences were tested using independent-samples *t*-tests; age and grade differences were tested using one-way ANOVAs. *t*-values are reported as absolute values. *p < 0.05, **p < 0.01, ***p < 0.001.

### 4.2 Common method bias assessment

Because all variables were derived from self-report questionnaire data collected from the same participants at a single time point, Harman’s single-factor test was used as a preliminary diagnostic check for common method bias. All measurement items were entered simultaneously into an unrotated exploratory factor analysis using principal component extraction. Six factors with eigenvalues greater than 1 were extracted, and the first unrotated factor accounted for 26.70% of the total variance. This value was below the commonly used 40% heuristic threshold in Harman’s single-factor testing (Tang and Wen, 2020; Chen et al., 2025), suggesting that no single factor dominated the item variance under this preliminary diagnostic criterion. In addition, a CFA-based model comparison was conducted. For the CFA-based common method bias diagnostic comparison, an item-level four-factor model with the four study constructs specified separately showed acceptable fit, *χ*^2^ = 2074.840, df = 521, *χ*^2^/df = 3.982, RMSEA = 0.040, CFI = 0.946, and TLI = 0.942, whereas the one-factor CFA model, in which all 34 items were loaded onto a single common factor, showed poor fit, *χ*^2^ = 15378.939, df = 527, *χ*^2^/df = 29.182, RMSEA = 0.123, CFI = 0.482, and TLI = 0.448. These results suggested that the measurement structure was not adequately represented by a single common factor. However, these diagnostic checks cannot fully rule out common method bias because all variables were self-reported, obtained from the same participants, and collected at a single time point (Podsakoff et al., 2024).

### 4.3 Correlation analysis of perceived usefulness of generative AI for learning, metacognitive self-regulation, academic procrastination, and generative AI dependency

Table 2 shows that PUGAIL was negatively correlated with MSR (*r* = −0.267, *p* < 0.001) and positively correlated with both AP and GAID (*r* = 0.324 and 0.314, respectively, both *p* < 0.001). GAID was negatively correlated with MSR (*r* = −0.273, *p* < 0.001) and positively correlated with AP (*r* = 0.338, *p* < 0.001), whereas MSR was negatively correlated with AP (*r* = −0.291, *p* < 0.001). Overall, this pattern of correlations was consistent with the hypothesized directions and provided a descriptive basis for the subsequent regression and PROCESS analyses of statistical indirect effects.

**Table 2:** Means, standard deviations, and correlations among study variables.

Variable	M	SD	1	2	3	4
Perceived usefulness of generative AI for learning (PUGAIL)	3.54	0.69	1			
Metacognitive self-regulation (MSR)	3.36	0.66	−0.267***	1		
Academic procrastination (AP)	3.63	0.69	0.324***	−0.291***	1	
Generative AI dependency (GAID)	3.46	0.61	0.314***	−0.273***	0.338***	1

*N* = 1869. Pearson correlations (two-tailed) are reported. ***p < 0.001.

### 4.4 Testing the statistical indirect effects of metacognitive self-regulation and academic procrastination in the association between perceived usefulness of generative AI for learning and generative AI dependency

**Figure 2:**
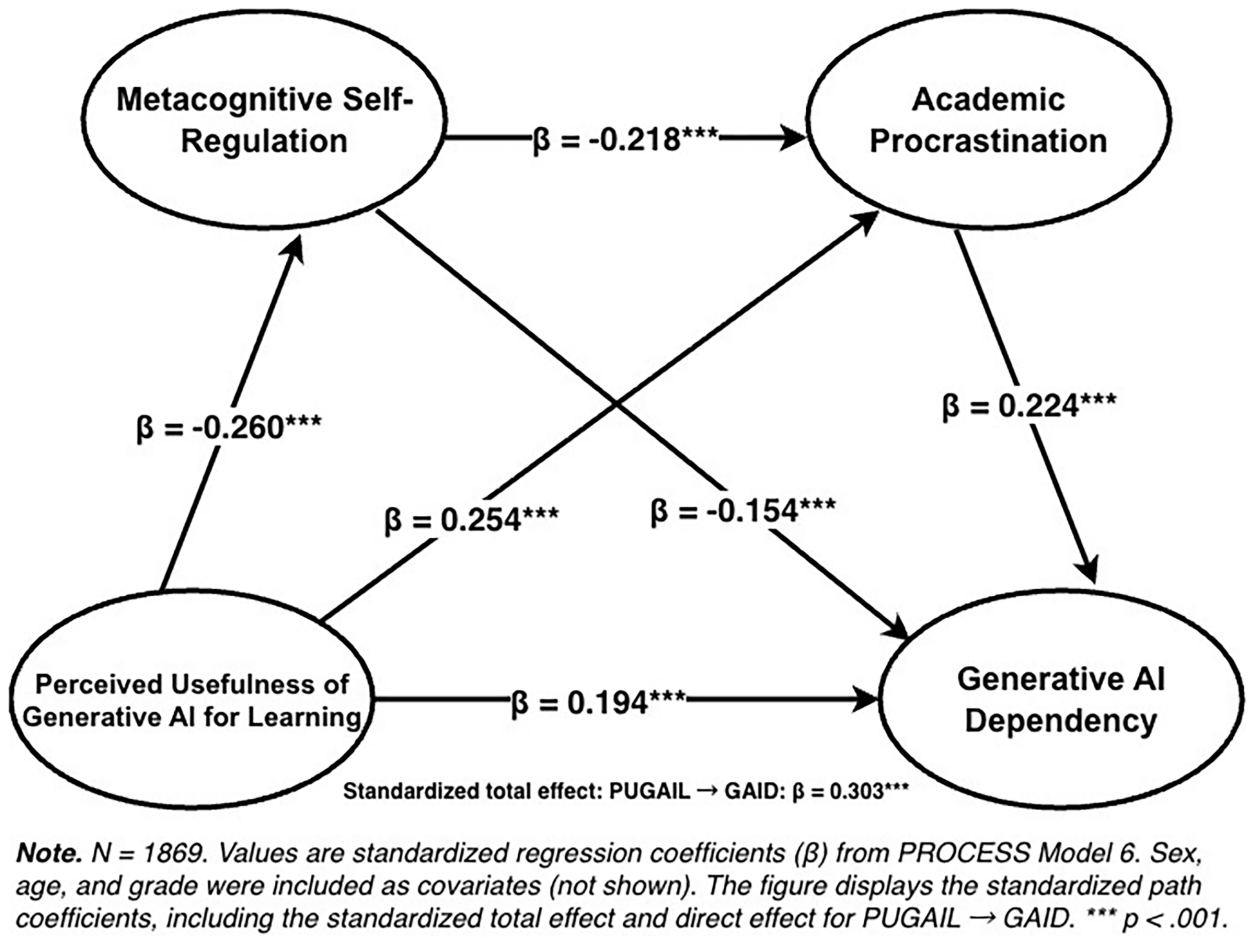
Standardized path coefficients for the PROCESS Model 6 statistical indirect effect model. Values are standardized regression coefficients (β). Sex, age, and grade were included as covariates but are not shown. The figure displays the estimated standardized path coefficients, including the standardized total association and direct association for PUGAIL → GAID. Because the data are cross-sectional, the paths represent statistical associations rather than temporal or causal effects. ***p < 0.001.

With perceived usefulness of generative AI for learning as the predictor variable, metacognitive self-regulation and academic procrastination as statistical mediators, and generative AI dependency as the outcome variable, PROCESS Model 6 (bootstrap = 5,000; *N* = 1,869) was used to estimate the specific and serial statistical indirect effects after controlling for sex, age, and grade. The prespecified four-construct measurement model used for measurement evaluation showed good fit in AMOS, with *χ*^2^/df = 1.427, RMSEA = 0.015, CFI = 0.992, and TLI = 0.992. Table 3 and Figure 2 the accompanying figure present the estimated path coefficients. Based on standardized path coefficients, PUGAIL was negatively associated with MSR (*β* = −0.260, *p* < 0.001) and positively associated with AP (*β* = 0.254, *p* < 0.001), whereas MSR was negatively associated with AP (*β* = −0.218, *p* < 0.001). In the equation with GAID as the dependent variable, MSR was negatively associated with GAID (*β* = −0.154, *p* < 0.001), whereas AP was positively associated with GAID (*β* = 0.224, *p* < 0.001). After MSR and AP were entered simultaneously, the direct association between PUGAIL and GAID remained significant (*β* = 0.194, *p* < 0.001), and the total association was also significant (*β* = 0.303, *p* < 0.001). The explained variance (*R*^2^) was 0.084 for MSR, 0.158 for AP, and 0.185 for GAID, whereas the *R*^2^ of the total association model was 0.105. All model F tests were significant (all *p* < 0.001).

**Table 3:** Regression estimates for the statistical indirect effect model.

Variable	Metacognitive self-regulation (MSR)		Academic procrastination (AP)		Generative AI dependency (GAID)		Overall effect	
	*β*	*t*	*β*	*t*	*β*	*t*	*β*	*t*
Perceived usefulness of generative AI for learning (PUGAIL)	−0.260	11.614***	0.254	11.434***	0.194	8.568***	0.303	13.712***
Metacognitive self-regulation (MSR)	—	—	−0.218	9.802***	−0.154	6.847***	—	—
Academic procrastination (AP)	—	—	—	—	0.224	9.811***	—	—
*R* ^2^	0.084		0.158		0.185		0.105	
*F*	42.980***		69.791***		70.261***		54.666***	

Standardized coefficients (*β*) are reported. Sex, age, and grade were included as covariates in all models (not shown). *t*-values are reported as absolute values. ***p < 0.001.

Table 4 further shows that the unstandardized total effect estimate was c = 0.267, 95% CI [0.2288, 0.3052], and that the direct effect estimate remained significant after MSR and AP were included, c’ = 0.171, 95% CI [0.1316, 0.2098]. The 95% confidence intervals for all three specific statistical indirect effects excluded zero. The statistical indirect effect through MSR was 0.035 (13.16%), 95% CI [0.0236, 0.0479]; the statistical indirect effect through AP was 0.050 (18.74%), 95% CI [0.0374, 0.0635]; and the serial statistical indirect effect from PUGAIL to MSR to AP to GAID was 0.011 (4.17%), 95% CI [0.0078, 0.0150]. The total statistical indirect effect was 0.096 (36.07%), 95% CI [0.0787, 0.1150]. Overall, the results were consistent with the directional expectations of Hypotheses 1–4. Although the serial statistical indirect path was significant, its relative contribution was modest. Given the cross-sectional design, these findings should be interpreted as statistical associations and statistical indirect effects rather than evidence that temporal ordering has been established or that causal mechanisms have been confirmed.

**Table 4:** Bootstrap estimates of statistical indirect effects and related effect-size indicators.

Path	Unstandardized estimate (B)	Proportion of total effect estimate	95% CI	
			LL	UL
PUGAIL → MSR → GAID	0.035	13.16%	0.0236	0.0479
PUGAIL → AP → GAID	0.050	18.74%	0.0374	0.0635
PUGAIL → MSR → AP → GAID	0.011	4.17%	0.0078	0.0150
Total indirect	0.096	36.07%	0.0787	0.1150
Direct effect (c′)	0.171	—	0.1316	0.2098
Total effect (c)	0.267	—	0.2288	0.3052

Estimates are unstandardized (B). Statistical indirect effects were estimated using percentile bootstrap 95% confidence intervals based on 5,000 resamples. Proportions of the total effect estimate were computed as (statistical indirect effect/total effect estimate) × 100%. Sex, age, and grade were included as covariates. Completely standardized statistical indirect effects (βCS) were: total indirect = 0.109 [0.0897, 0.1296], via MSR (PUGAIL → MSR → GAID) = 0.040 [0.0269, 0.0545], via AP (PUGAIL → AP → GAID) = 0.057 [0.0427, 0.0718], and via MSR → AP (PUGAIL → MSR → AP → GAID) = 0.013 [0.0089, 0.0171]. Given the cross-sectional design, these estimates should not be interpreted as causal effects.

## 5 Discussion

The present study examined the association between Perceived Usefulness of Generative AI for Learning (PUGAIL) and Generative AI Dependency (GAID) among university students from three higher education institutions in Changde, Hunan Province, China, as well as the statistical indirect roles of Metacognitive Self-Regulation (MSR) and Academic Procrastination (AP). Overall, the results showed a pattern of partial statistical indirect effects. The total statistical indirect effect accounted for 36.07% of the total effect estimate, whereas the direct effect estimate accounted for 63.93%. Among the statistical indirect paths, the path through AP accounted for 18.74%, the path through MSR for 13.16%, and the serial statistical indirect path from PUGAIL to MSR to AP to GAID for 4.17%. This pattern suggests that the procrastination-related path accounted for the largest proportion among the statistical indirect estimates, whereas the serial statistical indirect path played a more modest supplementary role. Given the cross-sectional and self-report design, these findings should be interpreted as statistical associations and statistical indirect effects rather than as evidence that temporal ordering has been established or that causal mechanisms have been confirmed.

### 5.1 Perceived usefulness of generative AI for learning and generative AI dependency

After controlling for sex, age, and grade, the total association between PUGAIL and GAID was positive (*β* = 0.303, *p* < 0.001), and the direct association remained positive after MSR and AP were entered simultaneously (*β* = 0.194, *p* < 0.001). This pattern suggests that students with more favorable evaluations of the usefulness of generative AI for learning were also more likely to report higher GAID. Previous research has similarly shown that higher levels of GenAI or ChatGPT use tend to co-occur with adverse outcomes such as academic procrastination, memory loss, and poorer academic performance (Abbas et al., 2024). This pattern is consistent with the possibility that when efficiency gains become a central rationale for use, repeated reliance on the tool may coexist with convenience as well as potential opportunity costs for autonomous learning processes. Consistent with this view, research in GenAI contexts has also shown that perceived usefulness or performance expectations may coexist with reliance-related concerns (Liu, 2025; Huang and Wu, 2025). This interpretation does not equate GAID with technology overuse or continuance intention; rather, it emphasizes dependency-oriented reliance in learning contexts.

From the perspective of the Technology Acceptance Model, perceived usefulness is a key cognitive belief underlying technology acceptance and continued use (Davis, 1989). When GenAI is experienced as saving time, improving output quality, and alleviating learning pressure, students may be more likely to incorporate it into routine learning strategies and show dependency-oriented reliance. Viewed through the lens of the I-PACE model, PUGAIL can also be understood as a form of outcome expectancy in learning contexts; such utility expectations may coexist with greater cue salience, more automated patterns of use, and difficulty reducing use, especially when GenAI provides immediate feedback and task-completion support (Brand et al., 2019). The fact that the direct association remained significant after MSR and AP were considered also suggests that other learning-process or dependency-related factors may still be involved. Dimensional research on AI dependency further indicates that it may involve emotional, functional, cognitive, and loss-of-control components that vary across measurement tools (Wu et al., 2026). Overall, the present findings suggest that higher perceived usefulness of generative AI for learning may coexist with higher dependency-oriented use.

### 5.2 Independent statistical indirect role of metacognitive self-regulation

The present study found a significant specific statistical indirect effect of MSR in the association between PUGAIL and GAID, consistent with Hypothesis 2. Specifically, PUGAIL was negatively associated with MSR (*β* = −0.260, *p* < 0.001), MSR was negatively associated with GAID (*β* = −0.154, *p* < 0.001), and the specific statistical indirect effect through MSR was 0.035, 95% CI [0.0236, 0.0479], accounting for 13.16% of the total effect estimate. This pattern suggests that stronger perceptions of GenAI as an efficient and labor-saving learning tool may coincide with lower metacognitive investment in planning, monitoring, checking, and reflection during use, and that lower MSR may coexist with stronger dependency-oriented use.

With respect to the PUGAIL–MSR association, trust-in-automation research suggests that when automated systems are perceived as effective and trustworthy, individuals may show monitoring complacency and automation bias, especially under conditions of higher task load or greater verification costs (Parasuraman and Manzey, 2010). This association can also be interpreted cautiously from the perspective of cognitive offloading: when learners judge that delegating work to a tool is faster, less effortful, and more likely to yield a usable answer, analysis, verification, and regulation that would otherwise be carried out internally may be shifted to the external system (Risko and Gilbert, 2016). In AI-use contexts, higher AI use has likewise been linked to stronger cognitive offloading, insufficient verification, and reduced engagement in higher-order cognition (Gerlich, 2025; Zhai et al., 2024), which is consistent with the higher PUGAIL–lower MSR association observed here.

With respect to the MSR–GAID association, the Strength Model of Self-Control suggests that when self-control resources or regulatory capacity are weaker, individuals are less able to consistently inhibit immediate, low-effort, and high-reward choices (Baumeister et al., 1998). In learning contexts, lower MSR may therefore correspond to weaker monitoring of use boundaries and may coexist with frequent help seeking, default invocation of the tool, and dependency-oriented use. Supporting evidence also shows that lower self-control or deficient self-regulation tends to co-occur with higher problematic technology use (Ding et al., 2022; Gökçearslan et al., 2016; Hrbackova et al., 2025). Taken together, the positive association between PUGAIL and GAID may be partly understood through a theory-informed statistical pathway linking higher perceived usefulness, lower metacognitive monitoring, and stronger dependency-oriented tendencies.

### 5.3 Independent statistical indirect role of academic procrastination

The present study also found a significant specific statistical indirect effect of AP in the association between PUGAIL and GAID, consistent with Hypothesis 3. Specifically, PUGAIL was positively associated with AP (*β* = 0.254, *p* < 0.001), AP was positively associated with GAID (*β* = 0.224, *p* < 0.001), and the specific statistical indirect effect through AP was 0.050, 95% CI [0.0374, 0.0635], accounting for 18.74% of the total effect estimate. This was the largest of the three specific statistical indirect effects, suggesting that AP may represent a behaviorally proximal statistical correlate in the association between perceived learning-related usefulness and dependency-oriented GenAI use.

This pattern is broadly consistent with related observations in previous GenAI research: more frequent or more dependency-oriented use of ChatGPT or GenAI in academic tasks tends to coexist with greater procrastination risk, and students’ positive perceptions of ChatGPT may coexist with concerns related to procrastination and ethics (Abbas et al., 2024; Uppal and Hajian, 2025; Bouzar et al., 2024). From the perspective of present bias or hyperbolic discounting, when GenAI is experienced as time-saving, effort-saving, and able to generate usable outputs quickly, learners may believe that they can still catch up later with the help of the tool. Such beliefs may coexist with delayed task initiation because the immediate psychological cost of starting the task becomes easier to defer (Steel, 2007). At the same time, procrastination may also be a relevant behavioral correlate of dependency-oriented use. Compensatory Internet Use Theory suggests that when pressure, guilt, and anxiety accumulate, individuals are more likely to turn to digital tools as a form of compensatory coping (Kardefelt-Winther, 2014). Related research has likewise examined procrastination as a mediator in the association between stress and internet addiction and has reported positive associations between technology dependency and procrastination across student samples (Gong et al., 2021; Chen and Lyu, 2024; Liu et al., 2022). Taken together, the positive association between PUGAIL and GAID may be partly understood through a theory-informed statistical pathway linking higher perceived usefulness, delayed task initiation, and stronger dependency-oriented tendencies.

### 5.4 Serial statistical indirect role of metacognitive self-regulation and academic procrastination

The present study further found a significant serial statistical indirect effect involving MSR and AP in the association between PUGAIL and GAID, consistent with Hypothesis 4. Specifically, PUGAIL was negatively associated with MSR (*β* = −0.260, *p* < 0.001), MSR was negatively associated with AP (*β* = −0.218, *p* < 0.001), and AP was positively associated with GAID (*β* = 0.224, *p* < 0.001). The serial statistical indirect effect was 0.011, 95% CI [0.0078, 0.0150], accounting for 4.17% of the total effect estimate. This pattern suggests that stronger perceived learning-related usefulness of GenAI may be associated not only with routine incorporation of the tool, but also with lower metacognitive monitoring, higher procrastination, and greater dependency-oriented use. This ordering is theoretically plausible because MSR reflects planning, monitoring, and strategy regulation, whereas AP reflects delayed task initiation and task execution; however, it should be interpreted as theory-informed rather than as evidence of temporal precedence or causal direction.

**Figure 3:**
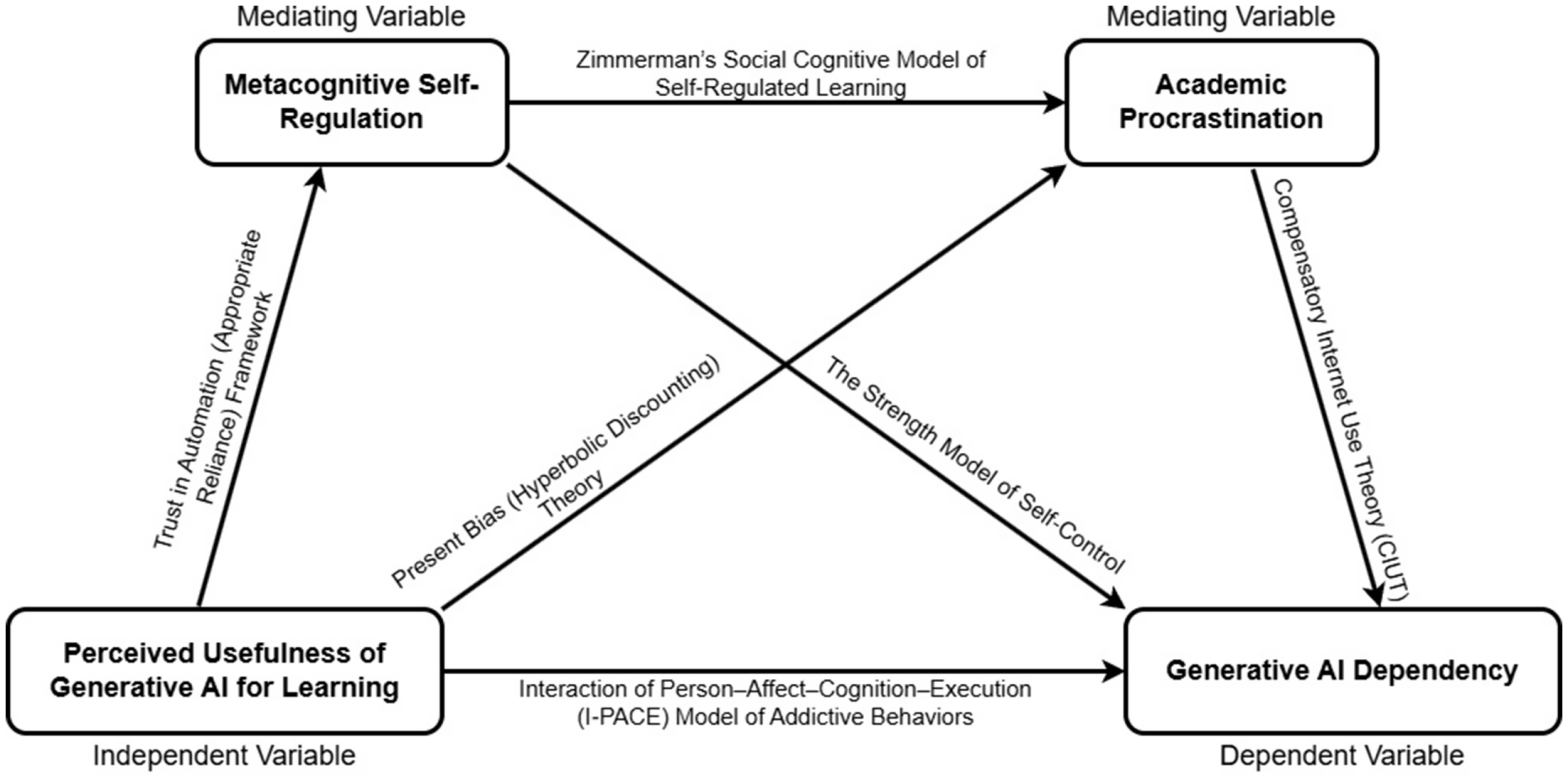
Theory-informed conceptual framework for the statistical indirect association model. The figure summarizes the theoretical perspectives used to specify the hypothesized links among perceived usefulness of generative AI for learning, metacognitive self-regulation, academic procrastination, and generative AI dependency. Arrows represent theory-informed statistical associations rather than established temporal or causal effects.

This statistical pattern is broadly consistent with prior discussions of cognitive offloading and self-regulated learning. When AI functions as an efficient and trusted external support, it may reduce immediate burden while also coinciding with lower introspection, reflection, or monitoring (Chirayath et al., 2025; Risko and Gilbert, 2016; Gerlich, 2025). At the same time, self-regulated learning research emphasizes that metacognitive regulation is closely tied to planning, monitoring, and task progression, such that weakened MSR is more likely to coexist with procrastination (Tao et al., 2025; Limone et al., 2020). From a further theoretical perspective, Compensatory Internet Use Theory suggests that when individuals experience pressure, guilt, or obstruction in real-world tasks, they are more likely to turn to digital tools for compensatory coping (Kardefelt-Winther, 2014). In academic contexts, the time pressure and need for last-minute remediation accumulated through procrastination may heighten the appeal of GenAI as a rapid means of coping with academic demands (Gong et al., 2021; Lardinoix et al., 2023). Accordingly, the serial statistical indirect path may be understood as a theory-informed statistical pattern linking cognitive-evaluative, regulatory, and behavioral levels (Figure 3). However, although this serial statistical indirect effect was significant, its relative contribution was limited, suggesting that it functions more as a supplementary statistical explanation of the association between PUGAIL and GAID than as a dominant pathway.

## 6 Practical implications

This study provides practical implications for teaching management and student support in higher education. By identifying a serial statistical indirect effect linking Perceived Usefulness of Generative AI for Learning, Metacognitive Self-Regulation, Academic Procrastination, and Generative AI Dependency, the findings suggest that favorable evaluations of GenAI for learning may coexist not only with stronger use tendencies, but also with weaker metacognitive monitoring, delayed task progression, and greater dependency risk. These findings highlight the dual-edged nature of GenAI use in learning and suggest that when universities integrate GenAI into teaching, they should attend not only to its value for efficiency, convenience, and task completion, but also to students’ self-monitoring, task management, and reflective use.

At the course and instructional level, instructors could consider process-oriented strategies around students’ learning processes. First, assignments, reports, papers, and project-based tasks could require process evidence such as outlines, drafts, revision notes, prompt records, and verification statements, which may reduce the likelihood that GenAI will be used as a direct substitute for the final product. Second, staged submission, milestone checks, and task decomposition may help reduce the tendency to postpone work until the deadline and then rely on GenAI for rapid last-minute remediation. Third, classroom activities and after-class training could incorporate information verification, source comparison, logical evaluation, and reflective explanation to support students’ metacognitive monitoring while using GenAI. Fourth, reasonable boundaries could be defined according to task type, encouraging the use of GenAI for information organization, idea generation, and structural refinement rather than for replacing independent analysis, argumentation, and critical judgment. The goal is not to deny the value of the tool, but to reduce the possibility that strong perceived usefulness gradually shifts into default invocation and habitual dependency.

At the institutional level, universities may incorporate AI literacy, time management, self-directed learning training, and academic integrity education into first-year orientation, learning support courses, and mental health services so that students can develop more mature standards for use and stronger self-management capacities. Institutions should also provide faculty with training in GenAI-oriented instructional design, promote a shift from purely product-oriented assessment to more process-oriented assessment, and gradually establish usage guidelines that vary by task type and course context. When classroom design, student support, and institutional norms are aligned, universities may be better positioned to preserve the learning-related benefits of GenAI while reducing the risks associated with insufficient monitoring, escalating procrastination, and dependency-oriented use. Because the present study was cross-sectional and observational, these practical implications should be regarded as theory-informed and data-informed suggestions rather than empirically validated interventions. Future experimental or intervention-based studies are needed to test whether these strategies can reduce dependency-oriented GenAI use and support students’ self-regulated learning.

## 7 Limitations and future directions

This study contributes to understanding the statistical associations and indirect effects among Perceived Usefulness of Generative AI for Learning, Metacognitive Self-Regulation, Academic Procrastination, and Generative AI Dependency. However, limitations related to self-report measurement and common method bias, cross-sectional design and causal inference, limited covariate adjustment, model robustness, and sample generalizability should be acknowledged. The present findings therefore require further examination through longitudinal, experimental, multi-source, and multi-region designs.

### 7.1 Limitations of measurement methods

This study relied primarily on self-report questionnaires administered to the same participants at a single time point. Accordingly, the findings may have been influenced by social desirability, subjective interpretation bias, same-source measurement, and common method variance. Although Harman’s single-factor test and the CFA-based model comparison suggested that the measurement structure was not dominated by a single common factor, these diagnostic checks cannot fully rule out common method bias, and the observed associations may still have been inflated. In addition, some scales were contextually adapted or used with modified response formats to fit generative AI-supported learning settings. Although these adjustments may have improved contextual relevance, they may also have reduced direct comparability with studies using the original scales. Future research could use time-lagged measurement, incorporate behavioral indicators such as platform usage logs, prompt records, and task-completion traces, combine self-reports with teacher ratings, peer ratings, or task performance, and further examine the stability and applicability of the adapted measures across different samples and contexts.

### 7.2 Limitations in study design and causal inference

This study used a cross-sectional questionnaire design and therefore can reveal only statistical associations consistent with the hypothesized theoretical ordering, rather than temporal precedence or causal direction. The PROCESS Model 6 analyses were intended to estimate statistical indirect effects rather than to confirm causal mechanisms. The associations may also operate in reverse or reciprocal directions. In addition, although the tested ordering was theory-driven, alternative orderings and competing models were not systematically examined. Future research could strengthen causal inference and test temporal ordering by using longitudinal, cross-lagged, or experimental designs.

### 7.3 Limited adjustment for confounding factors

Although sex, age, and grade were controlled in the regression and PROCESS models, the findings may still have been influenced by potentially important confounding factors that were not measured or controlled, such as AI literacy, learning motivation, academic stress, prior academic performance, course task difficulty, teachers’ norms regarding AI use, and the actual frequency and intensity of generative AI use. These factors may be associated simultaneously with perceived usefulness of generative AI for learning, metacognitive self-regulation, academic procrastination, and generative AI dependency, thereby influencing parameter estimates. Therefore, the observed associations and statistical indirect effects should not be interpreted as fully adjusted estimates or as evidence of causal relations. Future studies could incorporate a broader range of covariates and combine them with longitudinal or multilevel designs to improve internal validity and robustness.

### 7.4 Model robustness and hypothesis testing

Although this study evaluated the four-construct measurement model through confirmatory factor analysis and tested the hypotheses using regression and PROCESS analyses, evidence for model robustness remains limited. More specifically, beyond the CFA-based diagnostic comparison used to assess common method bias, the study did not systematically compare broader competing measurement models, alternative serial ordering models, or other structural specifications, nor did it conduct extensive sensitivity analyses or robustness checks. Therefore, the current results mainly reflect statistical support for the prespecified theory-informed model rather than evidence that this model is the only plausible specification. In addition, because questionnaires were distributed with the assistance of teachers from selected classes, responses may not have been fully independent at the class level. As no clustering adjustment was applied in the present analyses, standard errors and significance tests may have been affected by possible class-level clustering. Future research could improve the stability and replicability of the findings by incorporating competing model comparisons, tests of alternative serial orderings, multi-group robustness analyses, and multilevel or cluster-robust approaches.

### 7.5 Sample and generalizability

This study was based on data from university students enrolled in three higher education institutions in Changde, Hunan Province, China. Although the sample was relatively large and included both undergraduate and postgraduate students, it was still drawn from a single city. Thus, the sample should not be regarded as nationally representative of all university students in China. The findings may therefore have been shaped by contextual factors such as regional culture, institutional type, course requirements, and the local environment of generative AI use. Accordingly, caution is needed when generalizing the present findings to other universities, regions, cultural contexts, or age groups. Moreover, because measurement invariance was not systematically tested across sex, age, or grade groups, the observed group differences should be interpreted cautiously. Future research could replicate the study across multiple regions, different types of institutions, cross-cultural contexts, and broader student populations to improve external validity.

### 7.6 Future research directions and applications

Future research could proceed in several directions. First, longitudinal, cross-lagged, or experimental designs could be used to examine the temporal ordering and dynamic associations among Perceived Usefulness of Generative AI for Learning, Metacognitive Self-Regulation, Academic Procrastination, and Generative AI Dependency. Second, time-lagged measurement, usage logs, learning traces, process-based task records, and multi-source evaluations could be incorporated to improve measurement objectivity and mitigate common method bias. Third, variables such as AI literacy, actual frequency and intensity of generative AI use, prior academic performance, learning motivation, academic stress, course task difficulty, academic self-efficacy, and teachers’ norms regarding AI use could be included to examine alternative models, boundary conditions, and differences in association patterns. Fourth, replication studies across multiple regions, different types of higher education institutions, cross-cultural contexts, and broader student populations, together with further tests of model robustness, alternative serial orderings, and measurement invariance, would help clarify the stability and generalizability of the findings. Fifth, the present findings could inform course design, process-oriented assessment, and student support interventions, and future experimental or intervention-based studies could evaluate whether these strategies reduce dependency-oriented generative AI use and support students’ self-regulated learning.

## 8 Conclusion

Based on a sample of university students from three higher education institutions in Changde, Hunan Province, China, this study examined the associations between perceived usefulness of generative AI for learning and generative AI dependency, as well as the corresponding statistical indirect effects involving metacognitive self-regulation and academic procrastination. More favorable evaluations of the usefulness of generative AI for learning were associated not only with higher generative AI dependency, but also with lower metacognitive self-regulation and higher academic procrastination. Metacognitive self-regulation and academic procrastination were further involved in specific and serial statistical indirect effects within this association. These findings suggest that perceived usefulness of generative AI in learning contexts may coexist not only with convenience in tool use, but also with weaker learning monitoring, delayed task progression, and greater dependency risk. By integrating cognitive evaluation, learning regulation, and learning-related behavioral tendencies within a single analytic framework, this study provides a perspective for understanding the coexistence of convenience and dependency in generative AI use in higher education and offers data-informed implications for process-oriented instructional design, reflective-use training, and support for students’ self-directed learning.

However, because this study used a cross-sectional questionnaire design and relied primarily on self-report data from three higher education institutions in one city, the findings should be understood as statistical associations and statistical indirect effects consistent with the theoretical framework rather than as evidence that temporal ordering has been established or that causal mechanisms have been confirmed. Future research could use longitudinal, cross-lagged, or experimental designs and multi-source behavioral data to further examine the stability, boundary conditions, and dynamic processes underlying the relations among perceived usefulness of generative AI for learning, metacognitive self-regulation, academic procrastination, and generative AI dependency. Replication across different regions, types of higher education institutions, and broader student populations would further strengthen the external validity and practical relevance of this theory-informed framework.

## Data Availability

The original contributions presented in the study are included in the article, further inquiries can be directed to the corresponding author.
